# Annealing and passivation study of germanium on silicon (GOS) mid-infrared waveguide for sensing applications

**DOI:** 10.1038/s41598-026-35766-1

**Published:** 2026-02-02

**Authors:** Rachel C. F. Ang, Jia Sheng Goh, Landobasa Y. M. Tobing, Leh Woon Lim, Amy S. K. Tong, Andrew W. K. Fong, Zhixian Chen, Doris K. T. Ng

**Affiliations:** Institute of Microelectronics, Agency for Science, Technology and Research (A*STAR), 2 Fusionopolis Way, #08-02, Innovis Tower, Singapore, 138634 Republic of Singapore

**Keywords:** Germanium on silicon, GOS, Atomic layer deposition, Annealing, Passivation, Mid-infrared, Waveguide, Propagation loss, Materials science, Nanoscience and technology, Optics and photonics, Physics

## Abstract

Germanium on silicon (GOS) is an excellent platform for non-dispersive infrared sensing (NDIR) due to its broad mid-infrared (mid-IR) transparency. However, its optical waveguide propagation loss as a photonic sensing medium and its susceptibility to oxidation are concerns to be addressed. Herein, we study the effect of annealing GOS waveguide devices under forming gas and passivation using atomic layer deposition (ALD) of aluminum oxide (Al_2_O_3_) and aluminum nitride (AlN) on waveguide loss. Our findings showed that annealing helped reduce propagation loss as high as 17x at wavelength of ~ 5.85 μm and passivation with AlN was effective in minimising oxidation of germanium (Ge) in ambient, albeit at the expense of higher waveguide loss originating from the MIR absorption in the AlN film itself. Nevertheless, these results provide insights towards improving the performance and robustness of a GOS waveguide-based sensor.

**Supplementary Information:**

The online version contains supplementary material available at 10.1038/s41598-026-35766-1.

## Introduction

Optical chemical or gas sensing leverages on light-matter interaction^1–4^. Among the various optical sensing techniques, non-dispersive infrared sensing (NDIR) detects the drop in transmitted infrared (IR) radiation due to wavelength-specific absorption (molecular fingerprint) of the analyte when interacting with an infrared radiation source^5–12^. The miniaturization of NDIR sensors enhances portability, enabling mass deployment in various applications for instance, air quality monitoring^13^ in areas like commercial buildings and smart horticulture^14^ or gas leak detection^15^ in industrial settings to safeguard the health and safety of people and the environment.

One way towards miniaturization is via compact on-chip photonic waveguides. Various materials have been considered for mid-IR optical waveguides, including silicon, germanium and chalcogenide glasses with the choice of material used dependent on the sensing wavelength of interest^16–20^. Germanium on silicon (GOS) is an attractive platform for NDIR sensing due to its complementary metal-oxide semiconductor (CMOS) compatibility and broad mid-IR transparency range from 1.1 μm to 8.0 μm^21–23^ (limited by the silicon substrate). However, it is saddled with various loss mechanisms such as threading dislocation density (TDD) due to the lattice mismatch between the epitaxial germanium (Ge) layer and silicon (Si) substrate, surface scattering from the sidewall roughness induced from dry etch and absorption by the interstitial oxygen atoms in the Si lattice at wavelengths beyond 8.0 μm^24–26^. This results in wavelength dependent loss which peaks at around 9.3 μm^24^. GOS mid-IR waveguide devices have been demonstrated with propagation losses and wavelength ranges of ~ 3 dB/cm (λ = 2.3–3.8 μm)^27^, 2.5-5 dB/cm (λ = 6.70–7.45 μm)^28^ and 10 dB/cm (λ = 11 μm)^29^. Ensuring low propagation loss is imperative for sensing applications as interaction length of > 1 cm may be necessary for observable changes in the transmission signal for some applications^30^. As such, post processing techniques like hydrogen (H_2_) annealing has been widely studied to smoothen Si surfaces^31^. Likewise for Ge, thermal activation increases atomic mobility and hence atomic diffusivity allowing atomic migration, where it is observed that surface atoms tend to migrate from convex corners and settle at concave corners, thus smoothening the material surface^32^. Local laser annealing targeting waveguide sidewalls and non-local thermal annealing in hydrogen ambient have also been reported to yield promising results in the reduction of propagation loss^23^ by improving the surface roughness and passivating the surface dangling bonds^33^.

In addition to the various optical loss mechanisms in GOS waveguides, Ge is also prone to oxidation which limits the use of GOS in NDIR sensing. The presence of both humidity and oxygen (O_2_) in the environment creates conditions promoting oxidation of Ge^34^. Since Ge serves as the active sensing material, its oxidation and corrosion lead to alteration in its structural integrity, including surface roughness and composition, which can detrimentally impact the sensor performance and durability. To address this, studies have been conducted to passivate the Ge surface, such as using halogenic acids, which not only remove Ge oxides but also passivate Ge to minimize oxidation^35^,^36^.

In this paper, we fabricate GOS waveguides using 8-inch wafer level process and examine the effects of non-local thermal annealing in forming gas (4% H_2_ and 96% nitrogen (N_2_)), particularly on the propagation loss targeted towards improving gas sensors performance. The results show reduction in propagation loss as high as ~ 17x at λ = 5.85 μm post- annealing. We also explore atomic layer deposition (ALD) of aluminum oxide (Al_2_O_3_) and aluminum nitride (AlN) as passivation processes on GOS waveguides and observe that AlN appears to be more effective in preventing Ge oxidation compared to Al_2_O_3_. These results on GOS waveguides—with and without annealing, with different passivation approaches, and their impact on propagation loss—provide valuable insights into the behavior of GOS waveguides, guiding the development of high-performance and robust waveguide-based gas and chemical sensors.

## Results and discussion

### Effect of annealing on patterned ge film and mid-IR propagation loss

#### Formation of surface defects during thermal annealing process

The GOS waveguides are fabricated using 8-inch fabrication processes (see the Methods section). The annealed devices are examined under dark field microscope setting, scanning electron microscopy (SEM) and atomic force microscopy (AFM). Upon inspection, defects are observed on the Ge surface after annealing and its density and size are observed to be influenced by temperature and time which are supported by Kim et al.^37^ and Persichetti et al.^38^ As reported by Kim et al.^37^, the cause of the Ge surface defects stems from chemical desorption of volatile oxidized germanium monoxide (GeO) formed when reacting with O_2_ during annealing even under high vacuum condition. Apart from the chemical process contributing to the formation of surface defects, Persichetti et al.^38^ reported the formation and evolution of surface defects in Ge with thermal processes > 750 °C. Ge pits are formed due to atomic diffusion on the surface activated by thermal annealing whereby the enhanced surface mobility of Ge atoms promotes migration from high-strained region (near the embedded dislocations due to stacking faults in the bulk Ge) towards more relaxed area. As a result, net depletion of Ge atoms from strained areas forms the surface pit defects as observed.

#### Effect of temperature on defect density and size distribution

**Fig. 1:**
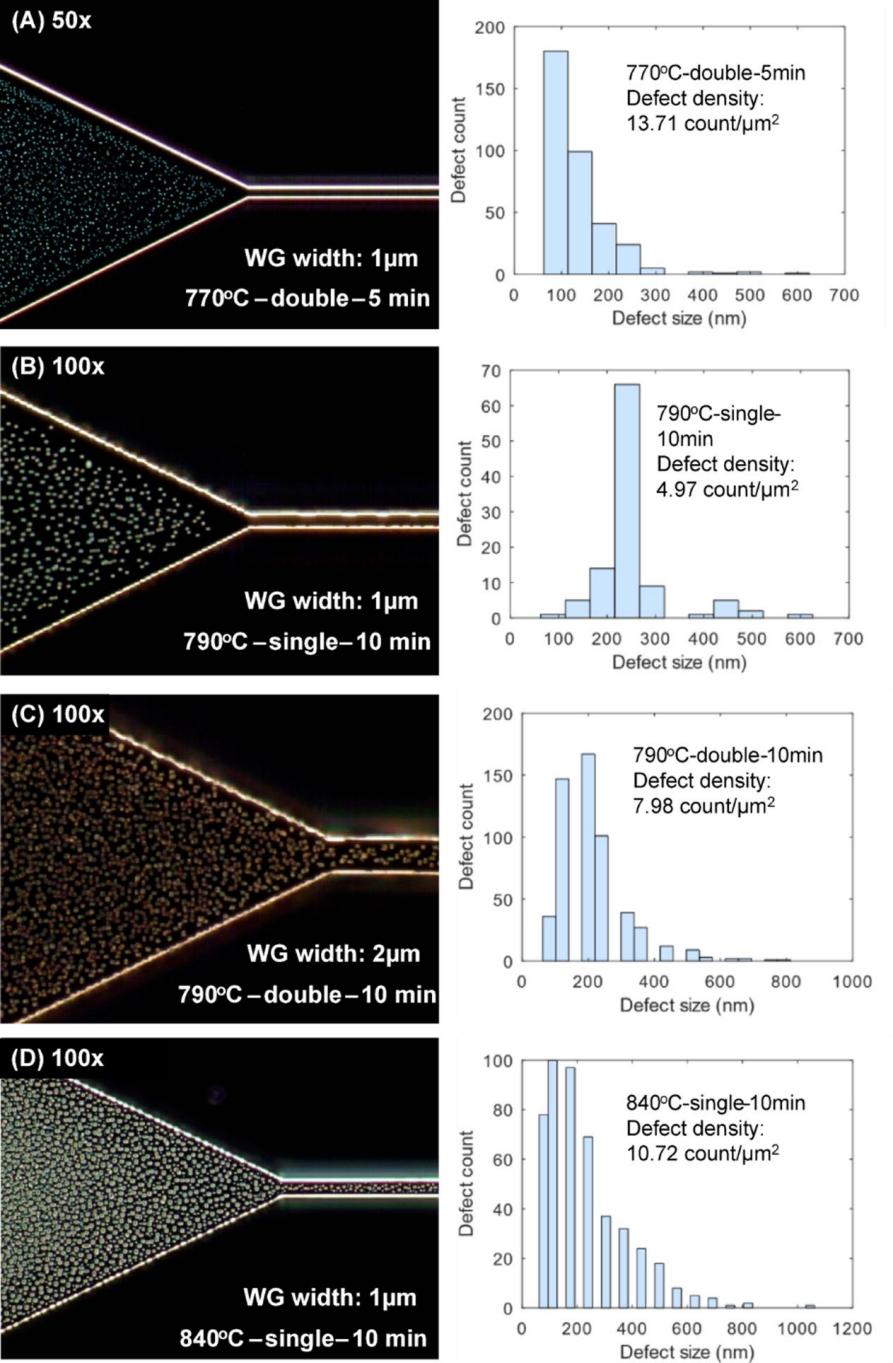
Dark field micrographs depicting defect size distributions on Ge surfaces when annealed at 770 °C, 790 °C and 840 °C for 5–10 min under forming gas.

The role of annealing temperature in the defect density and size was first studied in various annealing parameters, including the anneal temperature (770 °C, 790 °C, 840 °C), ramping scheme (single or double), and anneal duration (5 min and 10 min). In Fig. 1(A), it was observed that a lower annealing temperature and duration of 770 °C for 5 min, has the highest defect density of 13.71 counts/µm^2^ and smallest average defect size. For a fixed anneal duration of 10 min, further increasing annealing temperature from 790 °C to 840 °C introduces more surface defects, as observed in the increasing defect density from ~ 4.97 counts/µm^2^ to ~ 10.72 counts/µm^2^ [see Fig. 1(B)-(D)]. This also corresponds to the change of maximum defect size from 625 nm to 1062.5 nm, although their defect densities are not as high as in the case of annealing at 770 °C. This suggests an underlying activity relating to defect evolution to explain the larger defect size and lower defect densities observed as compared to Fig. 1A which will be discussed in Sect. 2.1.3.

#### Occurrence of the merging of defects and “landslide effect”

**Fig. 2:**
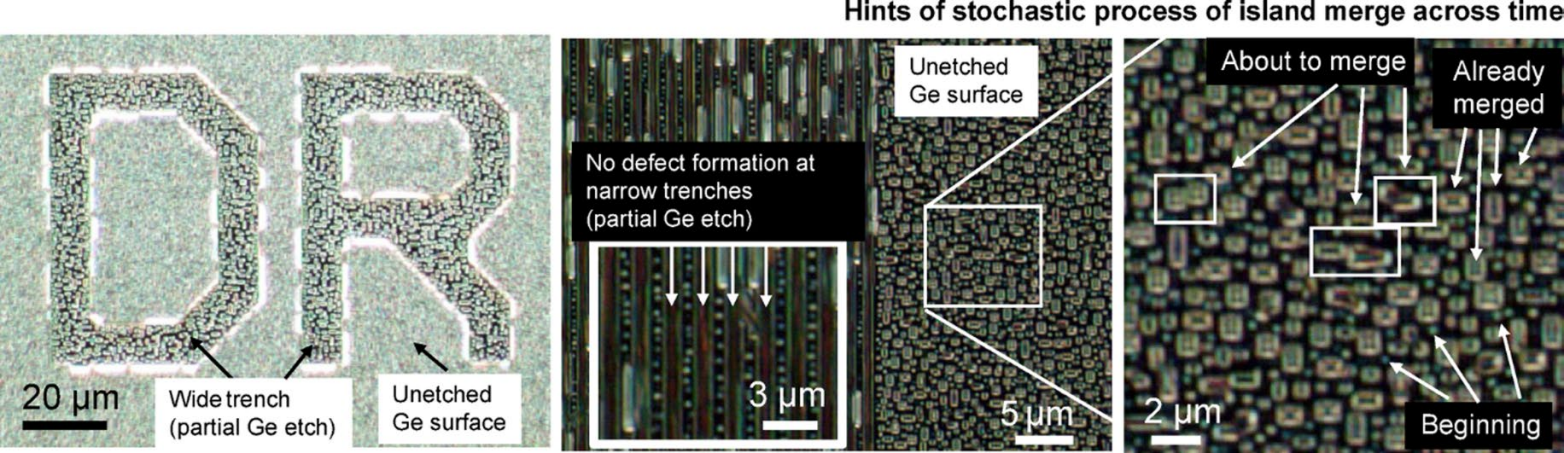
Defect formation on partially-etched Ge patterns. Dark field micrographs of GOS showing the growth of defects when annealed under forming gas at setpoint temperature 840 °C with single ramp for 10 min.

To further investigate the relation between defect sizes and annealing time/temperature, another annealing study was conducted on partially etched trench patterns (see Fig. 2). The initial stochastic process of defect formation was seen on Ge surface which began as a small defect. As annealing temperature and duration increases, the defect grew in size by seemingly merging with surrounding defects, resulting in the final defect size observed. Note that the formation of the defects also depends on the trench opening. When the trench is wide (Fig. 2, Left panel), defect formations were observed on both the top and bottom surface of the Ge. Here, the dark-field images focused on the bottom side of the Ge surface. When the trench width is in micron scale (Fig. 2, Center panel), no defect formation was observed within the trench. In addition, the merging of defects appears more pronounced in between the trenches, most likely because the movement is highly constrained in between the trenches. When we have a blanket area (Fig. 2, Right panel), we see similar defect distributions as those in Fig. 1. Such a coalescence of defects explained the phenomenon observed in Fig. 2, such that when Fig. 1A (770 °C, 5 min) is compared to Fig. 1C (790 °C, 10 min) the drop in defect density from 13.71 counts/µm^2^ to 7.98 counts/µm^2^ and the shift towards larger defect size was the outcome from the defects merging when annealing temperature and duration increased. This merging of defects was also observed by Persichetti et al.^38^.

**Fig. 3:**
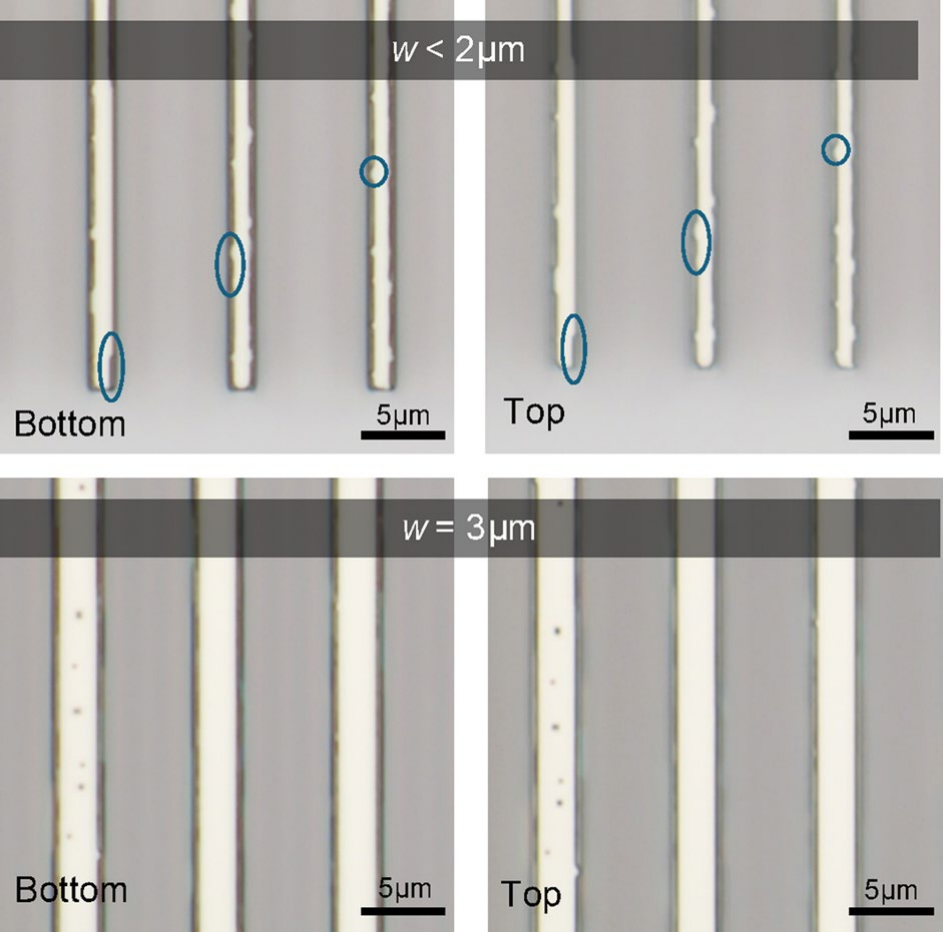
Bright field micrographs of fully-etched isolines with widths of 1.3 μm and 3 μm showing defect formation along the isoline edges.

Apart from the conjoined defects observed during defect merging at large structures (> 100 μm), another peculiar observation was the formation of jagged edges on isoline structures that are < 5 μm wide as seen in Fig. 3. This could be explained by high strain experienced along the edges compared to bulk regions where an enhancement in surface diffusion occurs from thermal stress buildup during annealing, thus forming large defects resembling a collapse at the edges resulting in this “landslide effect” phenomenon.

In addition, the occurrence of the jagged sidewalls of the isolines was observed across all waveguide widths given sufficient annealing time for defect formation. When the line width was increased to 3 μm, the emergence of surface defects on the Ge isolines occurred subsequently, showing the “landslide effect” within the isoline, thus the isoline width where the surface defect emerged was dependent on the annealing temperature and ramp condition. As a result of the significant annealing defects observed from double ramp process and long annealing duration, a further study on single ramp and shorter annealing duration was conducted (Supplementary Information, Fig. S1).

#### Study of single ramp annealing done with a shorter duration

**Fig. 4:**
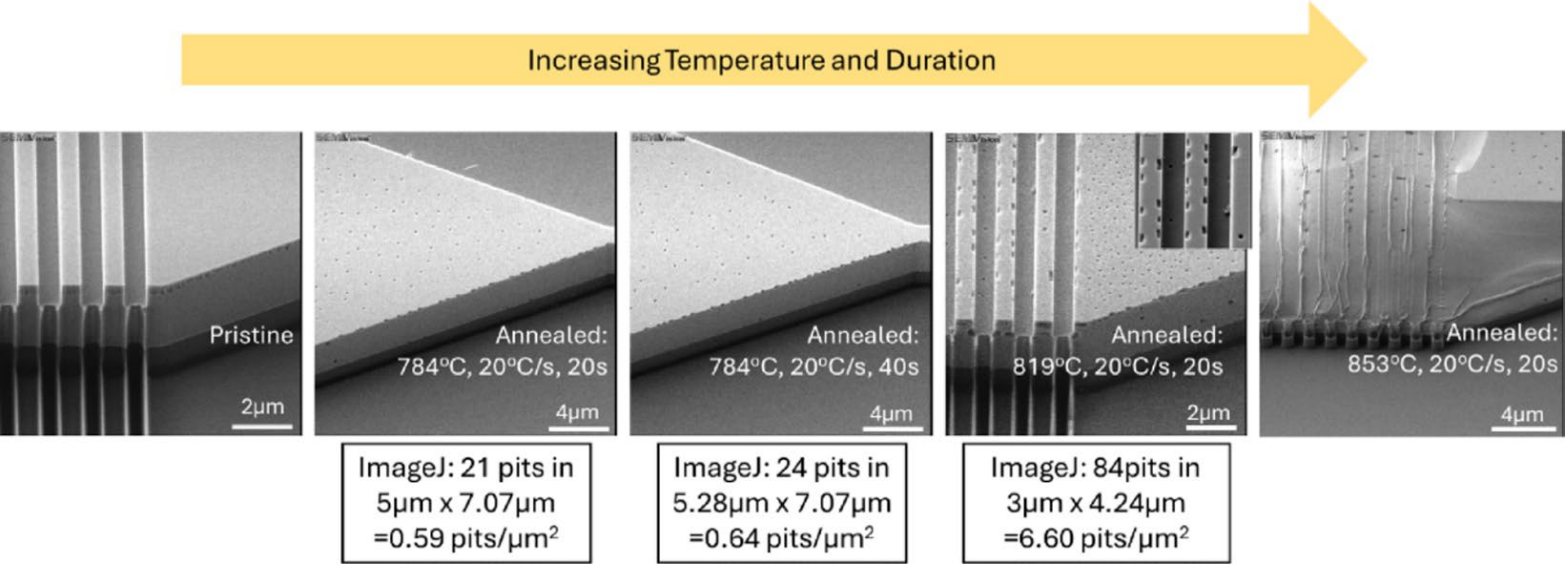
Oblique angle SEM images of fabricated GOS waveguides that underwent annealing at increasing temperature or duration starting with unannealed pristine device, thermal annealed at setpoint of 750 °C for 20 s and 40 s, 780 °C for 20 s and 820 °C for 20 s. However, due to an artefact we observed from our RTA process, the settling temperature for the annealing experiments were in fact at 784 °C, 819 °C and 853 °C from the thermocouple respectively.

To investigate the time dependence of defect formation, we conducted annealing experiments on GOS for short durations of 20 s and 40 s with the samples analyzed under SEM, AFM and ImageJ software^39^. From Fig. 4, it was observed that despite shortening the annealing duration from 10 min to 20 s, defects were still observed on Ge surface after annealing. These defects as seen from the SEM images were in fact pits on the Ge surface. Here, we observe the changes in pit density across the duration of 20 s to 10 min (Fig. 1). In contrast to Fig. 1, where the annealing was performed over several minutes, the annealing in Fig. 4 occurred on a much shorter timescale in a rapid thermal annealer (20 to 40 s). At this duration, the effect of coalescence is not evident. Instead, increasing the temperature from 784 °C to 819 °C accelerates the chemical desorption of volatile GeO, which saw an increase in pit density from 0.59 counts/µm^2^ to 6.60 counts/µm^2^. A further increase in temperature of > 850 °C had melted the Ge devices.

**Fig. 5:**
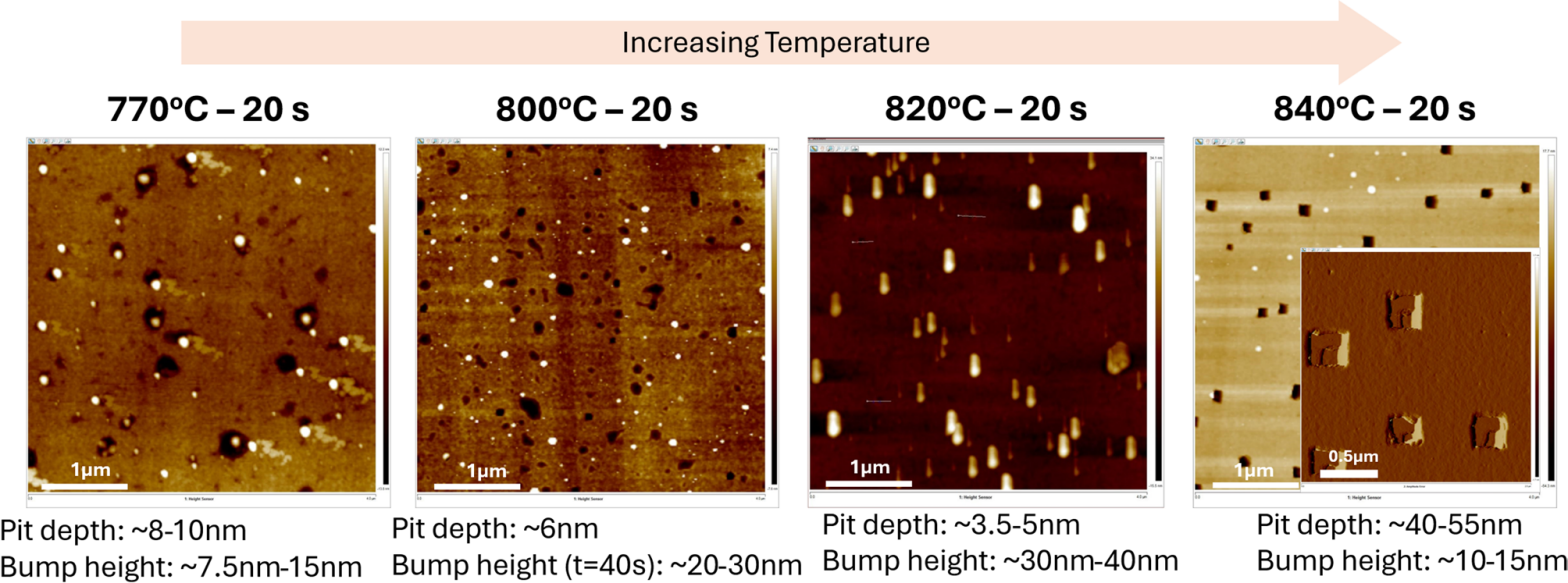
AFM images of 4 μm x 4 μm scan area of GOS surface after annealing for 20 s at 770 °C, 800 °C, 820 °C and 840 °C.

We also observed that the pits started to be more faceted at higher temperatures. Figure 5 showed that betweent 770 °C to 820 °C pits were about sub 10 nm in depth with varied shapes and sizes as measured with 2D atomic force microscopy (AFM). At > 820 °C, pits on Ge surface became more faceted with a square shape on top and got narrower towards the bottom resembling an inverted square base pyramid which was also reported by Kim et al.^37^. and Persichetti et al.^38^. Furthermore, as temperature increased from 770 °C to 840 °C, pit depths became more pronounced, increasing from 3.5 nm to 55 nm.

**Fig. 6:**
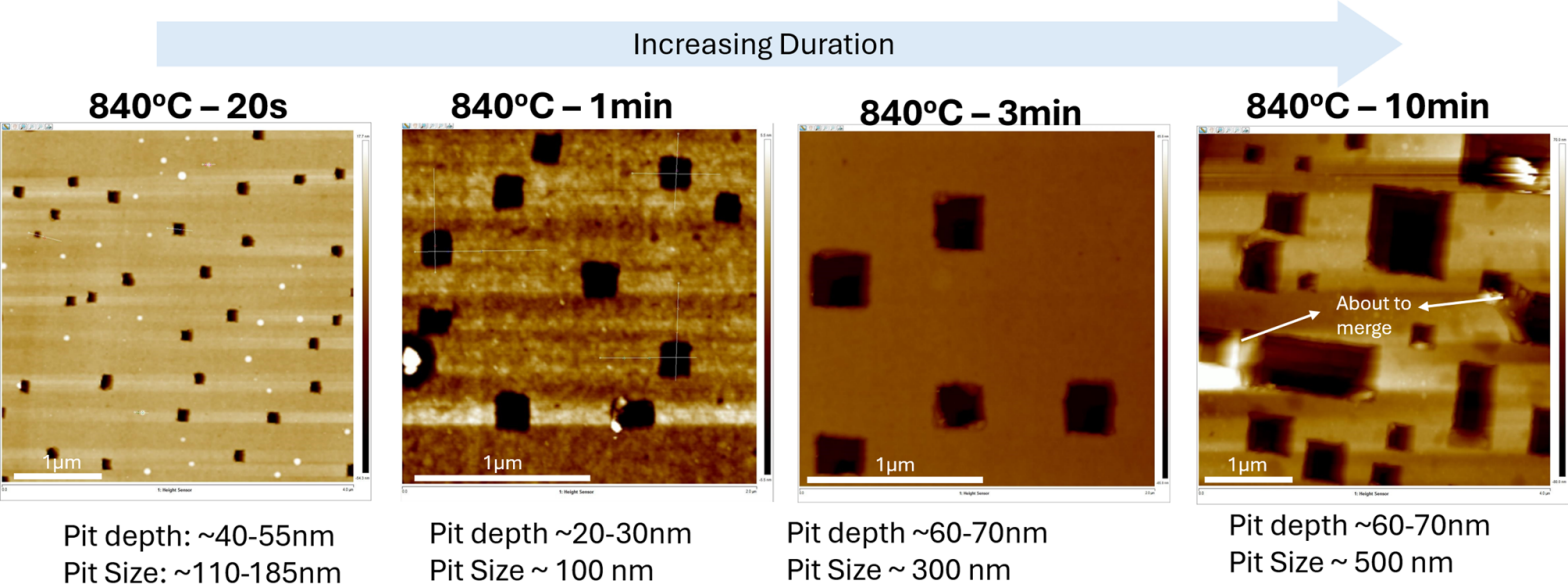
AFM images of GOS surface after annealing at 840 °C various duration of 20 s, 1 min, 3 min and 10 min.

According to Kim et al.^37^. , pit size and depth increased with annealing duration, which was also observed in Fig. 6. At a fixed annealing temperature of 840 °C with varying annealing duration from 20 s to 10 min, pit size grew from 100 nm to 500 nm and pit depth increased from 20 nm to 70 nm. It was also observed that pit depth was related to pit size, where larger pits have deeper recess. Similar to the dark field images in Fig. 2, pits in the process of merging can also be seen clearly from AFM images (see Fig. 6).

#### Influence of annealing temperature and duration on propagation loss

**Fig. 7:**
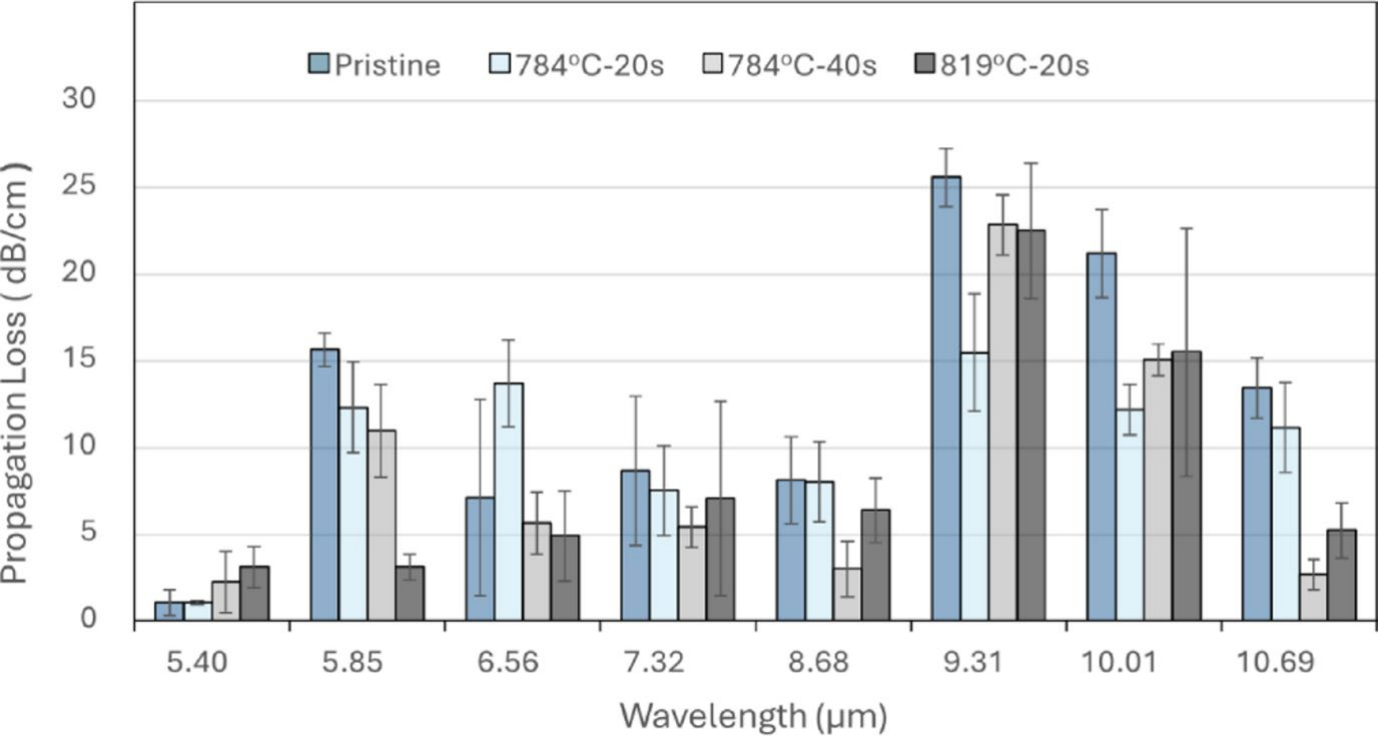
Measured propagation loss of waveguide on GOS chip annealed at 784 °C for 20 s and 40 s and 819 °C for 20 s as compared to pristine chip.

Propagation loss of annealed GOS waveguide devices was characterized from the cutback structures with waveguide lengths from 2 mm to 6 mm for 8 wavelength channels ranging from 5.4 μm to 11.0 μm of waveguide width from 1.1 μm to 3.0 μm using an in-house optical aligner setup (further detailed in the Methods section). The wavelength dependence in the propagation loss is evident, which is mainly attributed to the absorption from interstitial oxygen in the Si lattice at around 9.3 μm. We also observe other fluctuations in the measured waveguide loss due to the mixing of the TE and TM modes in our GOS waveguide. In our previous work^22^, the waveguide loss of TM and TE modes were simulated and found to be the upper and lower bound of our measured propagation loss (see Fig. S4. Supplementary Information). The results obtained in Fig. 7, showed that annealing improved propagation loss at longer wavelengths when compared to the pristine condition. The propagation loss for pristine chip ranges from 1.0 ± 0.7 dB/cm to 25.6 ± 1.7 dB/cm while for annealed chip at 784 °C for 20 s and 40 s it ranges from 1.0 ± 0.1 dB/cm to 15.5 ± 3.4 dB/cm and 2.2 ± 1.8 dB/cm to 22.9 ± 1.7 dB/cm respectively. Whereas annealed chip at 819 °C for 20 s, its propagation loss ranges from 3.1 ± 1.2 dB/cm to 22.5 ± 3.9 dB/cm. For all annealing conditions, the propagation loss peaked at 9.3 μm as expected from the mid-IR absorption from the interstitial oxygen in the Si lattice^25^,^26^. However, a significant reduction of loss by > 17x at 5.85 μm was measured where propagation loss decreased significantly from 15.65 dB/cm to 3.11 dB/cm when the chip was annealed at 819 °C for 20 s. The reduction in propagation loss at 5.85 μm could be attributed to the combined effects of forming gas annealing and moisture removal from the wafer, whereby the high-temperature anneal reduces infrared absorption associated with water bending vibrations near this wavelength^40^ leading to lower propagation loss. Therefore, despite the increase in pits on Ge surface caused by annealing, the overall propagation loss of annealed chips across the wavelengths improved even at 9.3 μm wavelength where the loss is the greatest due to absorption by interstitial oxygen in the Si lattice^25^,^26^.

### Oxidation of patterned GOS chip

**Fig. 8:**
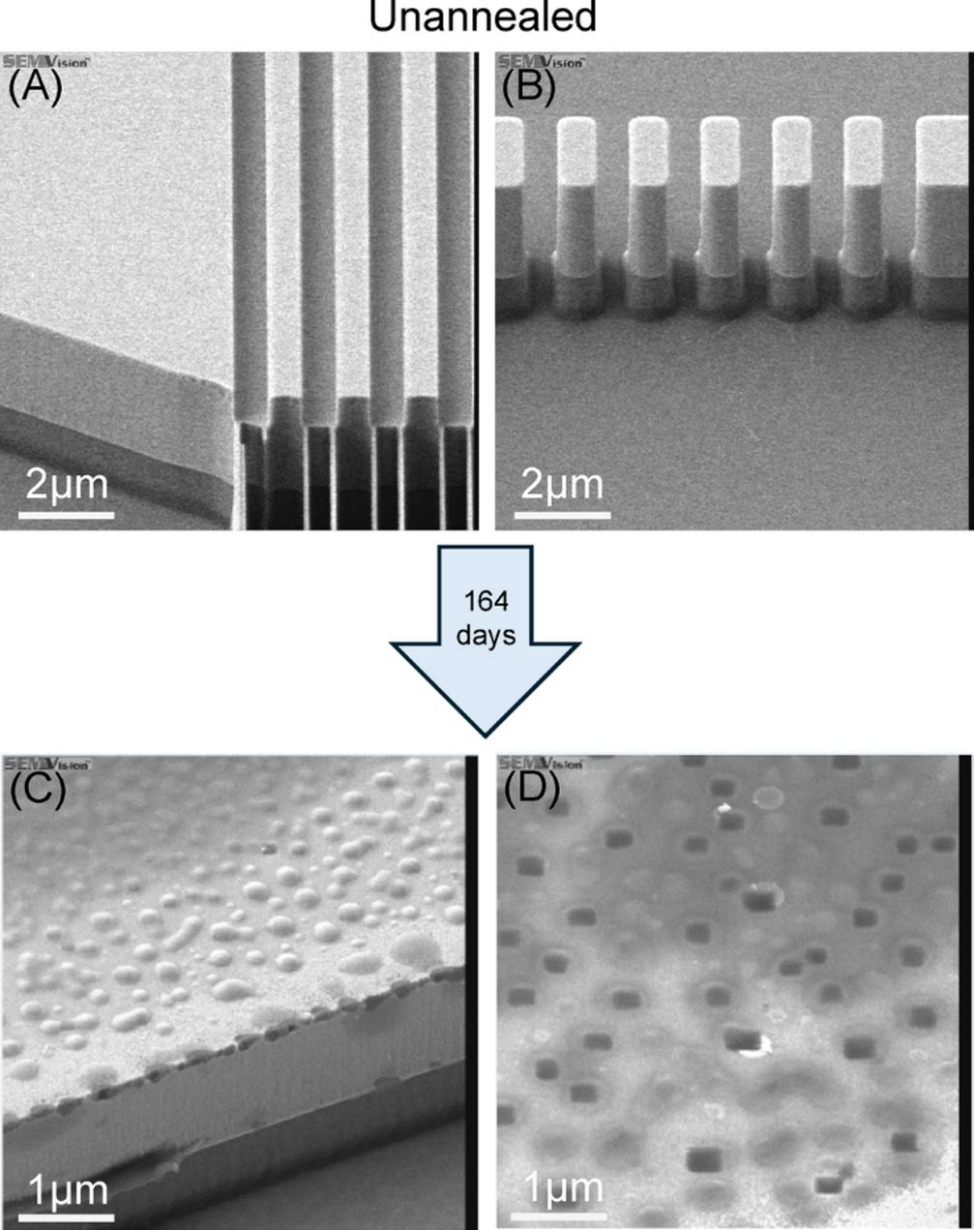
SEM images of GOS waveguide devices before and after being left in cleanroom condition for 164 days.

The formation of Ge pits is also observed when the GOS chip was left exposed to ambient cleanroom conditions of ~ 25 °C and ~ 40% humidity for > 5 months, where bumps appeared on the Ge surface, as shown in SEM micrograph in Fig. 8(C). Upon closer inspection, from another SEM detector view, the bumps resemble bubbles on top of pits on the Ge surface as seen in Fig. 8(D). Similar bubbles on Ge surface were also reported by Valladares et al. upon annealing of amorphous-Ge thin film on silicon dioxide (SiO_2_) due to outgassing of SiO_2_/Si substrate^41^. Therefore, the bubbles observed on our GOS chip could be a result of outgassing, but this does not explain the pits observed on the Ge surface. An oxidation study of monocrystalline porous Ge reported by Daniel et al.^34^. showed that humidity with O_2_ created a synergistic environment for rapid oxidation of Ge which was observed on our GOS chip. In Ref.^42^, it was reported that Ge oxidizes to form suboxides with increasing Ge oxidation number from Ge^1+^ (to form Ge_2_O) to Ge^2+^ (to form GeO) and Ge^3+^ (to form Ge_2_O_3_). Subsequently, a potential dissolution pathway of volatile Ge suboxides is via hydrolysis of Ge-O bonds due to the presence of ambient water, thus leaving behind surface defects in the form of pits (loss of Ge). The formation of these pits took much longer time than in the annealing case, due to the lack of thermally assisted processes. Lastly, the formation of the bubbles on the pits could be attributed to the combination of the oxidative process and outgassing. Over time, substrate outgassing continues in conjunction with the formation of Ge suboxides, as Ge forms high oxidation state Ge^4+^ that precipitated out as GeO_2_, bubbles are formed when the volatile gases (such as GeO, H_2_O etc.) are encapsulated. Therefore, when GOS chip was left under cleanroom condition, the ambient O_2_ and humidity created an environment for the continuous oxidation and dissolution of Ge suboxides forming pits on Ge surface and when combined with the outgassing of substrate and GeO_2_ precipitation, resulted in the formation of bubble artifacts on the pitted Ge surface.

### Effect of film passivation on propagation loss

Ge has a large transparency in the midIR range from 1.1 μm to 15 μm which coincides with the chemical fingerprint region, thus making it a suitable candidate for gas and chemical sensor devices. Yet, as evidenced in Fig. 8, Ge is highly susceptible to oxidation, prompting the need for passivation, to protect against oxidation and reaction with chemicals or gases, in the context of sensing application. Studies have shown that oxide and nitride films have been used to provide corrosion protection for metals in aggressive environments (e.g. aqueous, saline and oxidizing etc.) for industrial applications with promising results^42–45^. Therefore, in our study we explore various passivation layers, such as Al_2_O_3_ and AlN, deposited conformally by atomic layer deposition (ALD), to passivate the Ge film and improve corrosion resistance. The propagation loss of passivated GOS chips was then measured and compared with their pristine counterparts.

**Fig. 9:**
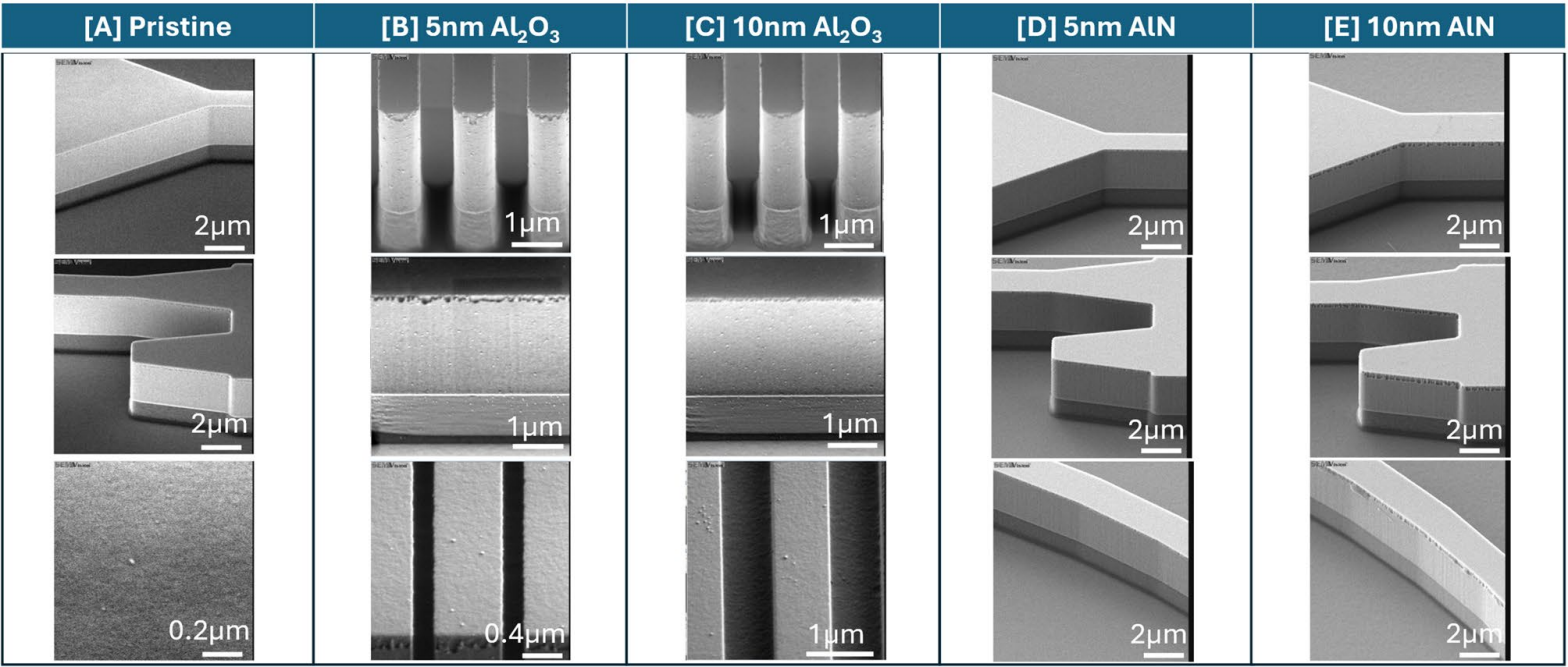
SEM images of pristine GOS chip and GOS chips as deposited with 5 nm and 10 nm Al_2_O_3_ and AlN by ALD.

**Fig. 10:**
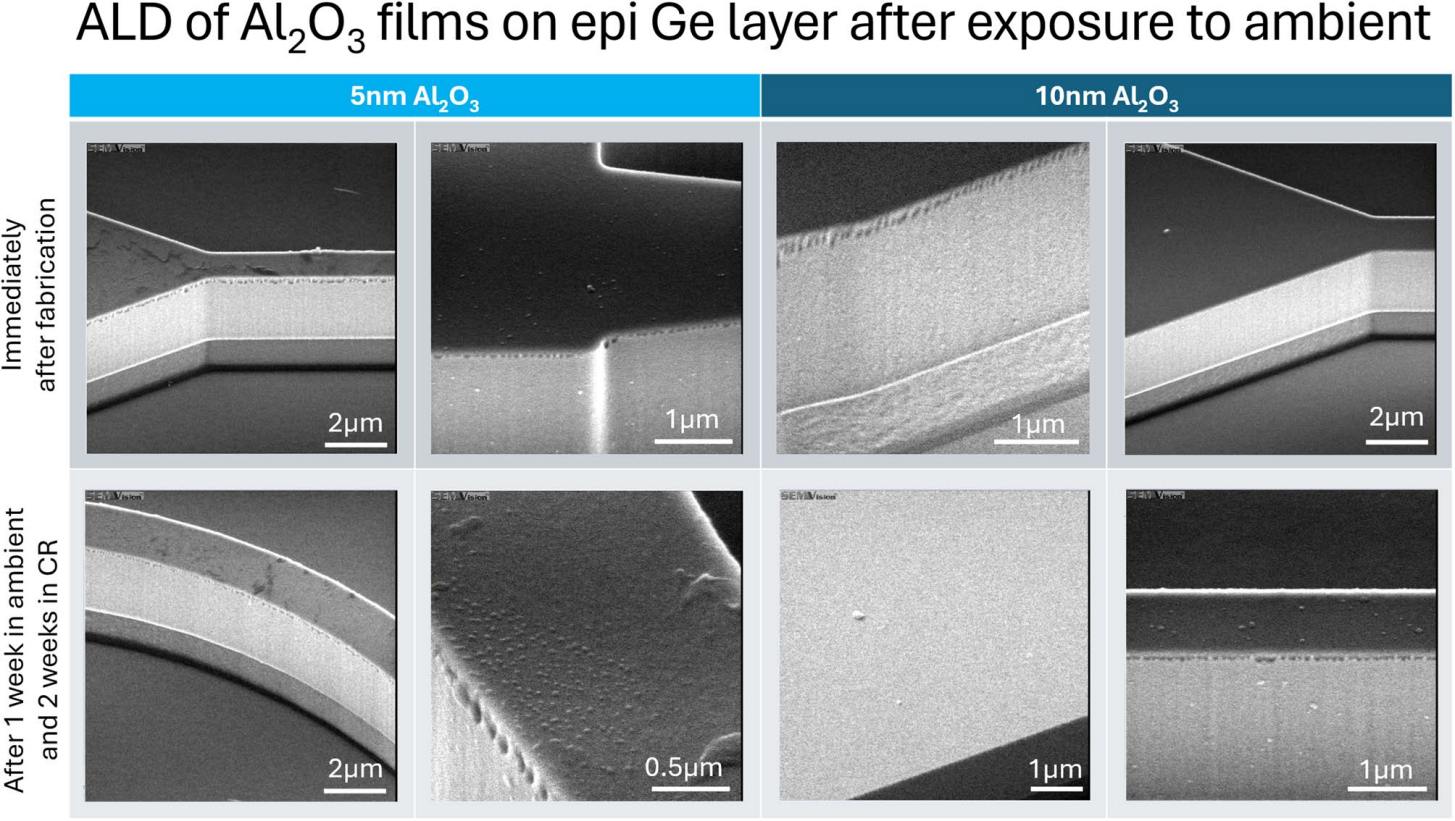
SEM images of as deposited 5 nm and 10 nm Al_2_O_3_ on GOS chips and after being left out in the ambient and cleanroom.

From Fig. 9, small bumps were observed on pristine GOS chip and as deposited 5 nm and 10 nm Al_2_O_3_ on GOS chips whereas GOS chips passivated with AlN remain smooth. To study the effectiveness of passivation with Al_2_O_3_ and AlN films in minimizing oxidation, the ALD deposited GOS chips were left in ambient and cleanroom environments. As seen in Fig. 10, small bumps were observed on as deposited Al_2_O_3_ films where the number of bumps were higher in the 5 nm than 10 nm Al_2_O_3_ deposited chips. Energy dispersive x-ray (EDX) spectroscopy was done at 5 kV on the bumps which showed major elemental signals of carbon (C), O, Ge, Al and Si as shown in Supplementary Information Fig. S2. It is possible that the H_2_O precursor used during the ALD process for Al_2_O_3_ introduced reaction pathways to oxidize the Ge surface.

**Fig. 11:**
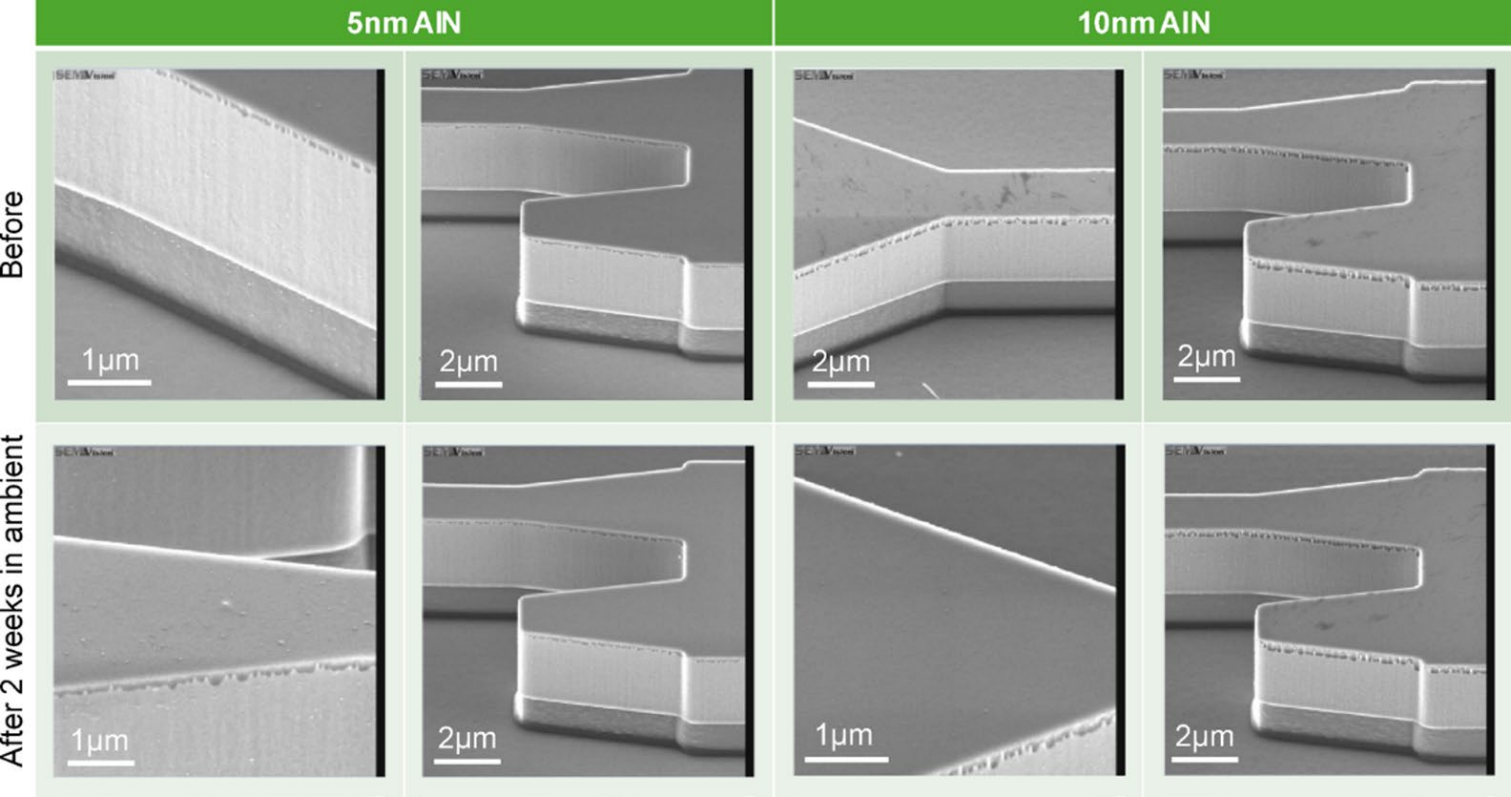
SEM images of as deposited 5 nm and 10 nm AlN on GOS chips and after being left out in the ambient.

As a result of the bumps observed even after passivating GOS chip with Al_2_O_3_, passivation with AlN was investigated to avoid the use of O-containing precursor gases. From Fig. 11, we observed that the passivated GOS surface remained generally smooth despite being kept in the ambient for 2 weeks, indicating AlN is more suitable than Al_2_O_3_ in passivating Ge surface against oxidation.

**Fig. 12:**
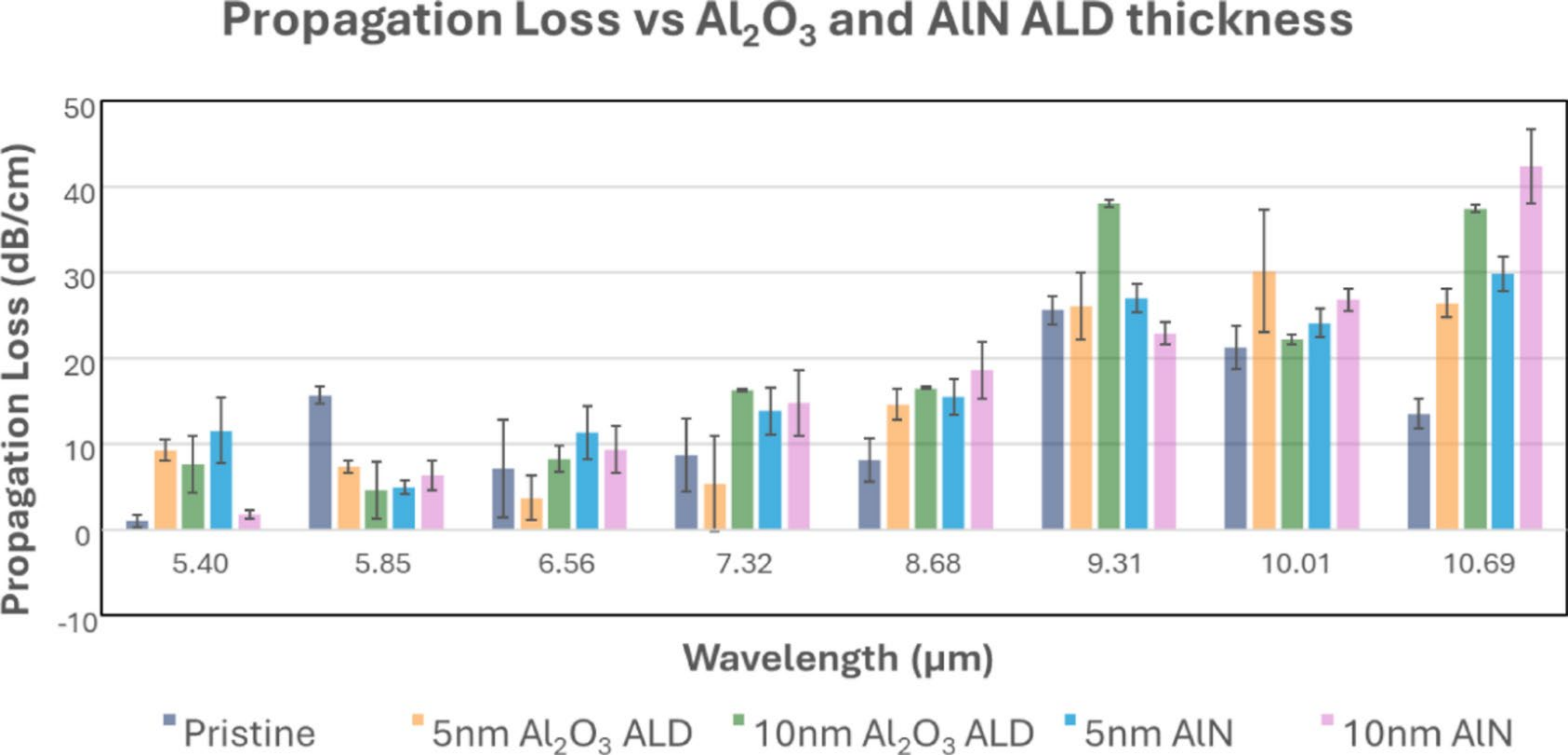
Measured propagation loss of waveguide on pristine GOS chip and GOS chips passivated with 5 nm and 10 nm Al_2_O_3_ and AlN.

To analyze the effect of Al_2_O_3_ and AlN passivation film on the propagation loss, waveguide loss measurements were conducted on the passivated GOS chips. As shown in Fig. 12, both 10 nm Al_2_O_3_ and AlN tend to have higher propagation losses than 5 nm Al_2_O_3_ and AlN. The loss also increases at longer wavelengths for both Al_2_O_3_ and AlN films, and this agrees with the wide band absorption at 10 to 20 μm of both Al-O bonds in Al_2_O_3_^46^ and Al-N bonds in AlN^47^. However, at 5.85 μm, both Al_2_O_3_ and AlN have at least 4x lower propagation loss than the pristine GOS chip. Comparing the propagation loss between Al_2_O_3_ and AlN films across the wavelength range, there is no clear distinction on which film is able to have a lower propagation loss as their effect on the propagation loss is wavelength specific. On the other hand, as we observed oxidation after Al_2_O_3_ ALD process, AlN may be a more suitable material for passivating GOS chips.

**Fig. 13:**
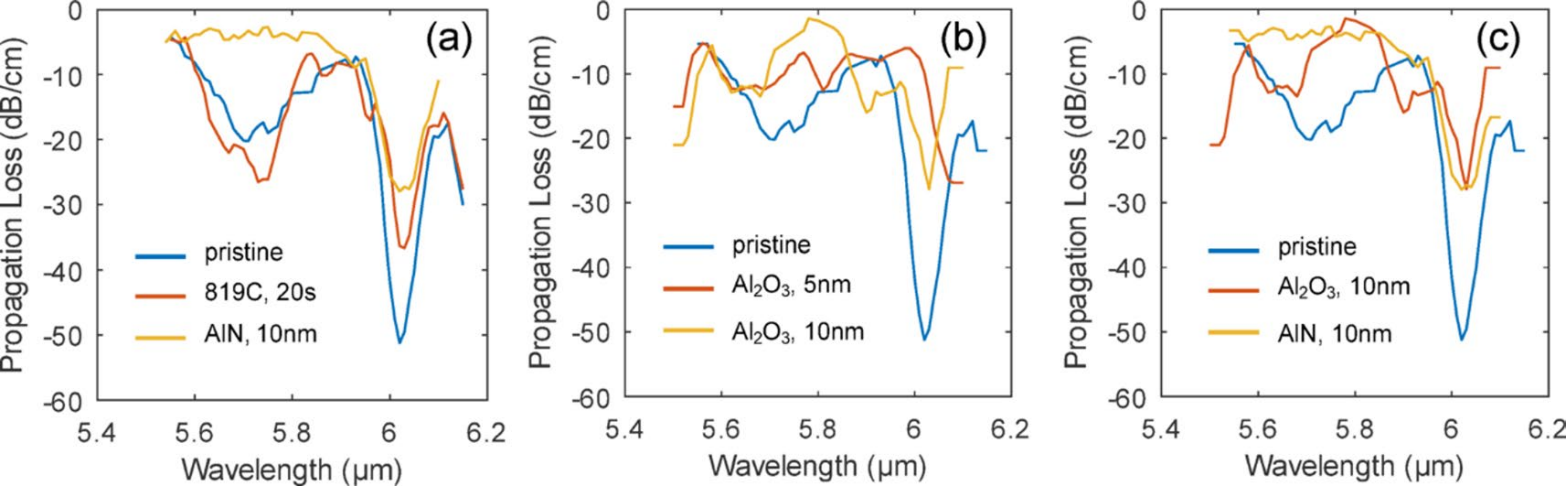
Propagation loss of GOS waveguide with various annealing and passivation condition around 5.85 μm.

The propagation spectra around 5.85 μm for GOS waveguide under various conditions are shown in Fig. 13. The two spectral dips in the loss spectrum are mainly attributed to the water absorption in the mid-IR spectrum (around 6 μm). After annealing the sample (819 °C, 20 s), we can see that the loss associated with water absorption decreases. However, after passivating the GOS waveguide with 10 nm thick AlN film, we can see further reduction at these spectral dips. The effect of coating thickness for Al_2_O_3_ coated samples is presented in Fig. 13b. Thinner coating generally leads to a lower propagation loss, despite some fluctuations which are likely caused by mixed polarizations in our loss measurement. The effect of different passivation layers is shown in Fig. 13c. AlN appears as a more stable passivation layer than Al_2_O_3_ in reducing water-related absorption. We believe this is related to the TMA and H_2_O precursors in the Al_2_O_3_ deposition, in contrast to the TMA and NH_3_ precursors in AlN deposition. Through our study on annealing and passivation, we believe that these could represent complementary strategies for mitigating loss in GOS based waveguides. Our results from annealing in forming gas showed a reduction in absorption losses at around 6 μm and at 10 to 11 μm as a result of the removal of moisture and reduction of Ge-O bonds through hydrogen termination. However, it does not provide lasting protection against reoxidation. In contrast, dielectric layer such as AlN act as a robust passivation barrier against subsequent oxidation, though it is unable to remove pre-existing oxidation states. Therefore, we believe that a combination of both approaches allows a reduction of absorption losses and enhanced stability compared to either method alone.

**Table 1:** Comparison of our work and previous research on the propagation loss of optical waveguides in the 3.29 μm to 7.70 μm wavelength range.

Waveguide Design	Waveguide Dimensions	Wavelength (µm)	Loss range (dB/cm)	Post treatment	Refs.
Ridge	2.0 μm thick GOS 3.3 μm wide, 1 μm partial etch	6.70–7.45	2.50–5.00	NA	^28^
Sub-wavelength grating suspended	1.0 μm thick Ge 2.7 μm wide, suspended 3 μm in air	7.70	5.20	NA	^48^
Suspended nanorib	5.2 μm thick Chalcogenide on 2 μm SiO_2_, 0.2 μm height x 0.2 μm wide, suspended 2.0 μm in air	3.291	4.5	NA	^16^
Strip meander	Chalcogenide on 2 μm SiO_2_, 0.3 μm height x 6 μm wide	3.291	3.6	Ag island nanostructure	^17^
Strip	3.0 μm thick GOS 4.0 μm height x 1.1 μm wide	5.40–7.32	1.04–15.65	NA	This work
			1.06–5.41	Annealing H_2_ (4%), N_2_ (96%)	
			1.80–5.38	Al_2_O_3_ or AlN film	

A comparison of our work and previous research on the propagation loss of GOS waveguides in the wavelength range of 5.40 μm to 7.70 μm can be seen in Table 1. From the table, we observed that our work has shown improvement in reducing the maximum propagation losses from up to 15.65 dB/cm to < 5.50 dB/cm for both annealing and ALD passivation treatments. Despite the differences in waveguide design and dimensions, the post treatments of our GOS strip waveguide were able to achieve propagation losses comparable to what had been reported by other researchers using more shallow GOS ridge waveguide^28^ and Ge subwavelength grating suspended waveguide^48^. Furthermore, the simplicity of our strip waveguide design that also leverages on both GOS platform and post-treatments (forming gas annealing or passivation with Al_2_O_3_ or AlN) enables a non-complex, CMOS compatible fabrication process that makes it feasible for large scale manufacturing.

## Materials and methods

### Device fabrication

The GOS waveguides used in this work are fabricated from commercially available 3 µm thick Ge-on-Si substrate. The Si substrate is an 8-inch, p-type boron doped, < 100 > crystal orientation with resistivity > 1 ohms-cm, while the Ge layer grown is undoped with a 3 µm +/- 5% thickness. Due to the film thickness, a backside coating is applied to reduce the wafer warpage followed by standard 8” wafer level processing tools such as deep-UV stepper lithography, inductively coupled plasma dry etching to define the grating couplers and channel waveguides. The fabricated wafers are diced and further processed on chip-level when investigating the effects of annealing and passivation by ALD.

### Annealing process in forming gas

**Fig. 14:**
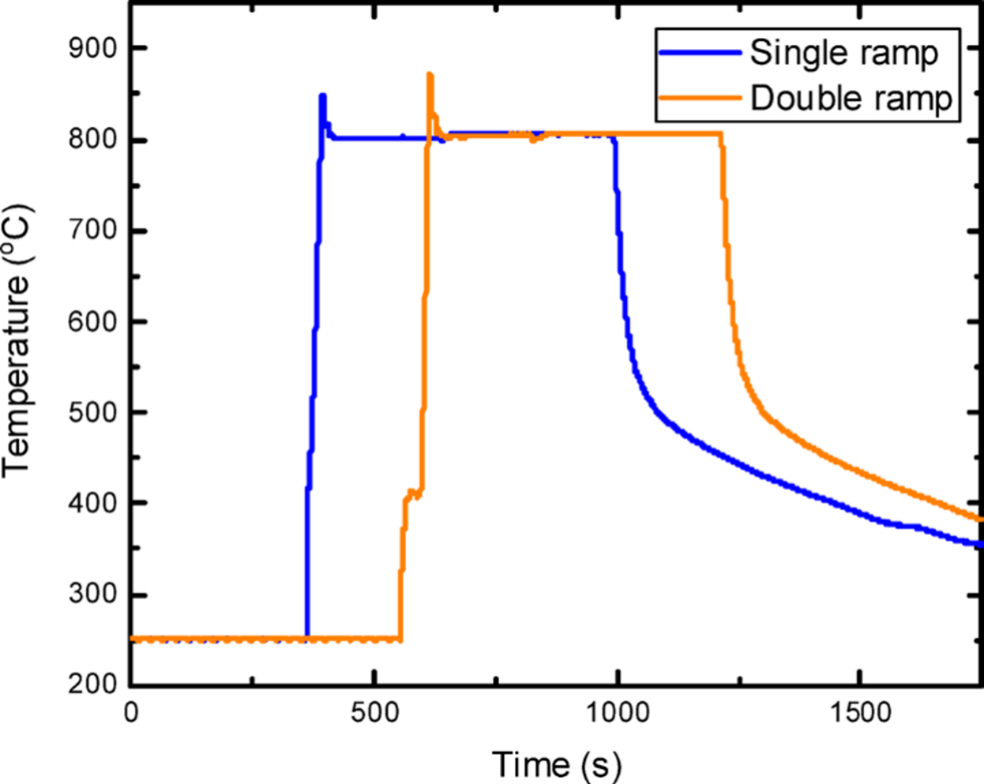
Plot of temperature (°C) against time (s) for GOS chip annealed at set temperature 840 °C for 10 min in a rapid thermal processor under forming gas.

For the annealing experiments conducted, diced GOS waveguide device chips are aenneald in a rapid thermal processor under forming gas (4% H_2_ and 96% N_2_). These are done at set temperatures ranging from 770 °C to 840 °C for 20 s to 10 min at a ramp rate of 20 °C /s with either single ramp or double ramp. For both ramp conditions, the chamber temperature is preset to 250 °C. In single ramp processes, samples are directly ramped up to the desired setpoint temperature, whereas in double ramp processes, samples are first ramped up to a set temperature of ~ 400 °C and held for 30 s before ramping up again to the desired set temperature. A plot of the temperature against time during the single and double ramp annealing process is shown in Fig. 14.

### Atomic layer deposition (ALD) of Al_2_O_3_ and AlN films

For the passivation study of GOS waveguide devices, Al_2_O_3_ or AlN films are deposited by ALD on diced GOS waveguide. To ensure conformal passivation of the Ge waveguides, the thickness of the passivation layer is designed to be at least 2–3x the sidewall roughness of the Ge waveguide, which we measure to be in the range of 2–3 nm by 3-dimensional (3D) AFM (shown in Fig. S3 and Table 2 under Supplementary Information). Based on this, 5 nm and 10 nm of Al_2_O_3_ and AlN films are deposited. For the Al_2_O_3_ deposition, we use trimethylaluminum (TMA) and water (H_2_O) as the precursor gases. To minimize oxidation of Ge surface during the ALD process, the deposition is done at a low temperature 150 °C and with increased TMA dose for the first few cycles to saturate the Ge surface and minimize the chance of H_2_O reacting with the Ge surface. For the ALD process of AlN, precursor gases of TMA and ammonia (NH_3_) are used under plasma at 375 °C.

### Waveguide measurement

**Fig. 15:**
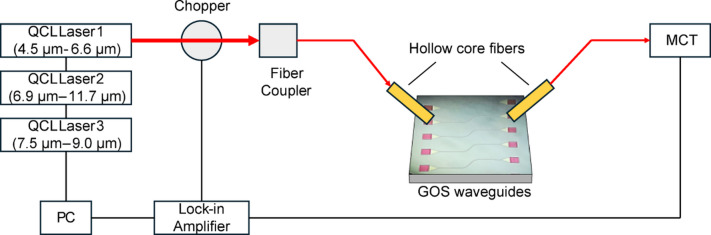
Schematic of the waveguide measurement setup that was used to measure propagation loss of cutback structures.

For both annealing and passivation studies, the GOS waveguide chip measured has 8 wavelength channels which ranges from 5.4 μm to 11 μm all fabricated within a single reticle. Propagation losses across the wavelength range are measured using cutback structures whose lengths vary from 2 mm to 6 mm with specifically designed grating couplers for each wavelength. Before conducting waveguide measurements of the GOS devices, a routine power check is done on the optical setup to adjust the focusing lens alignment for optimal power output measured by a power meter. The optical setup used comprises of a series of quantum cascade lasers (QCLs) that can emit mid-IR wavelengths ranging from 4.5 μm to 11.7 μm suited for our device requirements. The QCL used is modulated by a chopper before being guided by a hollow core fiber to the grating coupler on our chip. The received output is then guided by another hollow core fiber into a mercury cadmium telluride (MCT) photodetector for data collection aided by a lock-in amplifier. A schematic of the waveguide measurement set up is shown in Fig. 15 below. As the designed wavelength may differ from the final fabricated outcome due to variations in the grating critical dimension, a wavelength sweep is done to determine the center wavelength for each waveguide length of 2 mm to 6 mm, after which repeated measurements at the same wavelength is done with the peak voltage recorded to determine the propagation loss. The models of the QCLs used were MIRcat-QT-2100 (7.5 –9.0 μm), MIRcat-2300 (6.9 –11.7 μm), and MIRcat-2400 (4.5 –6.6 μm) from Daylight Solutions. These were guided by hollow fibers of part number HF500MW-FC-Gn-1 m from Guiding Photonics and finally detected by the MCT detector model 1427 C-AV from HORIBA Instruments Incorporated.

### Dark field imaging, SEM and AFM

A microscope with 50x-100x objective magnification lens is used to observe morphological changes of the GOS device surface with dark field mode enhancing the contrast. In addition, SEM is done at 45^o^ tilt angle, under small field of view and compared between the different SEM detectors. This provides a 3D view with higher magnification quality, enabling both the Ge surface and its sidewall condition to be closely examined. Furthermore, to quantify the defects observed, 2-dimensional (2D) AFM using the tapping mode is used on the GOS device surface to support our observations and gather more data on the surface defects such as the pit size and depth across a given scan area.

### Defect density and size distribution characterization

The characterization of etch defect density and size distribution of as-etched Ge and evolution of oxidative defects with annealing are performed using ImageJ^39^. From the image captured, a scale is set to relate pixel size to known distance. Enhancement of image contrast, brightness and sharpness is done followed by applying thresholding to ensure accurate representation of surface morphology for the detection of defects and their surface area.

## Conclusion

Signal strength, sensitivity and lifetime are important aspects in NDIR sensor. This work explores annealing and passivation in the attempt to reduce waveguide loss and protect Ge waveguides against unwanted oxidation for sensing applications. It has been found that annealing reduces waveguide loss up to 17x at 819 °C for 20 s. However, this loss reduction tends to be self-limiting as the size and density of the annealing-induced defects also increase with annealing time and temperature, resulting in higher scattering loss. In terms of passivation, AlN is shown to be a better passivation layer than Al_2_O_3_ in minimizing oxidation in ambient. However, this comes at the expense of higher waveguide loss, which originates from MIR absorption from the AlN passivation layer. Owing to this trade-off, a critical AlN thickness will be required to minimize propagation loss while remaining chemically inert against oxidation. Future work would include a combination of passivation-annealing schemes and polarization-resolved loss characterization on the GOS waveguides. Results obtained in this work provide insights for photonics waveguide designs and other applications in the mid-IR wavelength region in addition to improving GOS waveguide for gas and chemical sensor performance and lifetime.

## Supplementary Information

Below is the link to the electronic supplementary material.

Supplementary Material 1

## Data Availability

The data set generated or analyzed during the course of the study are available from the corresponding author upon reasonable request.
