# Supplementary material for: Annealing and passivation study of germanium on silicon (GOS) mid-infrared waveguide for sensing applications
Source: Sci Rep. 2026 Feb 2;16:6909. doi: 10.1038/s41598-026-35766-1 (PMC12917015; doi:10.1038/s41598-026-35766-1)
Supplement: Supplementary file 1 — Supplementary Material 1 [file 41598_2026_35766_MOESM1_ESM.docx]

Supplementary Information

Annealing and passivation study of germanium on silicon (GOS) mid-infrared waveguide for sensing applications

Rachel C. F. Ang^1^, Jia Sheng Goh^1^, Landobasa Y. M. Tobing^1,*^, Leh Woon Lim^1^, Amy S. K. Tong^1^, Andrew W. K. Fong^1^, Zhixian Chen^1^, Doris K. T. Ng^1^

^1^Institute of Microelectronics, Agency for Science, Technology and Research (A*STAR), 2 Fusionopolis Way, #08-02, Innovis Tower, Singapore 138634, Republic of Singapore

*Corresponding author: Landobasa Y. M. Tobing, email: Landobasa_Tobing@a-star.edu.sg

*Effect of ramp condition on defect density, size distribution and minimum surface area of defect onset*

As important as the effect that annealing temperature had on the GOS devices, the ramp condition also displayed a significant effect on it. In double ramp annealing process, the chamber initially at 250°C ramped up to ~400°C and held for 30 s before ramping up again to the desired temperature. Before reaching the desired temperature, a spike in temperature was recorded. The temperature spike in double ramp annealing is higher than single ramp annealing, as seen in Fig. 13 plot of the temperature against time during the annealing process.

With the temperature and duration kept constant at 790°C for 10 minutes, comparing Fig. 1B (single ramp) and Fig. 1C (double ramp), when annealing was done with double ramp, defect density increased from 4.97 counts/µm^2^ to 7.98 counts/µm^2^ with a longer distribution tail towards defect size. Moreover, as observed in Fig. S1, despite annealing at a lower temperature 790°C, the double ramp annealing showed a higher defect density and had the onset of defect occurring earlier at smaller feature width w>1.7µm (GC12), as compared to single ramp annealing done at a higher temperature of 840°C the defect onset occurred later at larger feature width w >2.0 µm (GC16). This could possibly be explained by the larger spike in the max temperature reached for double ramp process with an example of the process shown in Fig. 1, thus expediting the oxidative desorption and surface diffusion processes resulting in higher defect density and defect size. In fact, in Fig. 1, the double ramp process had the max temperature spiked up to 872°C as compared to 848°C in single ramp, and it was this critical increase of >20°C that had caused the GOS structure to melt (not shown here). Due to the uncontrollable larger spike that double ramp had on GOS devices, which may be detrimental to GOS devices, single ramp anneal was investigated to optimize the annealing process as discussed in section 2.1.4.


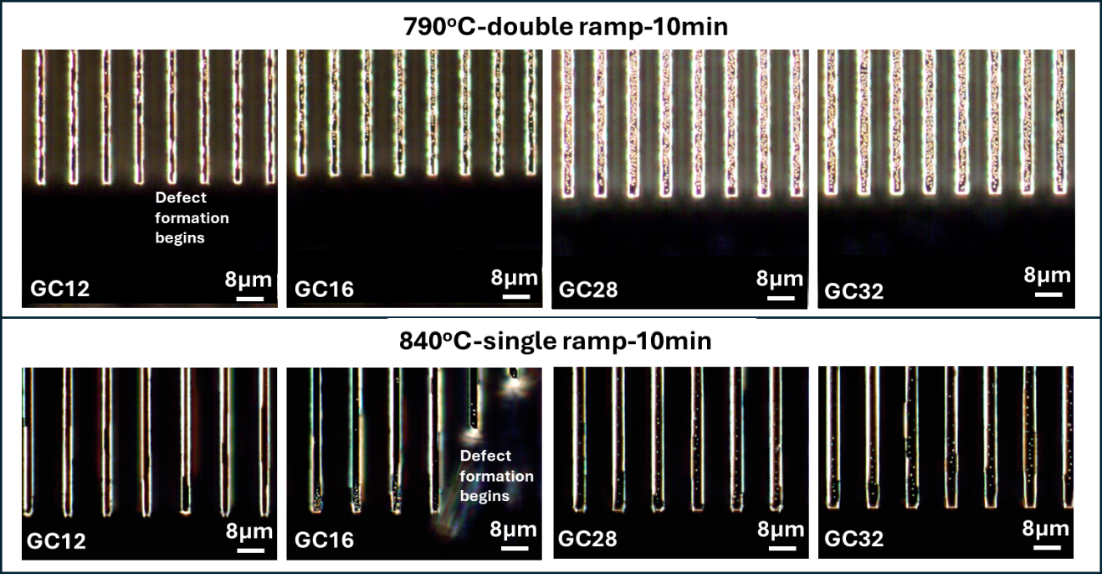


**Figure S1.** Dark field micrographs of GOS waveguides annealed under forming gas at set temperatures 790°C and 840°C with double and single ramp respectively for 10 minutes.

*Oxidation of GOS sample with Al_2_O_3_ after being kept in ambient.*

Figure S2 shows the SEM image and EDX spectrum of the bumps on the GOS sample which shows major elemental signal of C, O, Ge, Al and Si. The presence of the bumps and the Ge and O elemental signals suggests that Ge had oxidized.


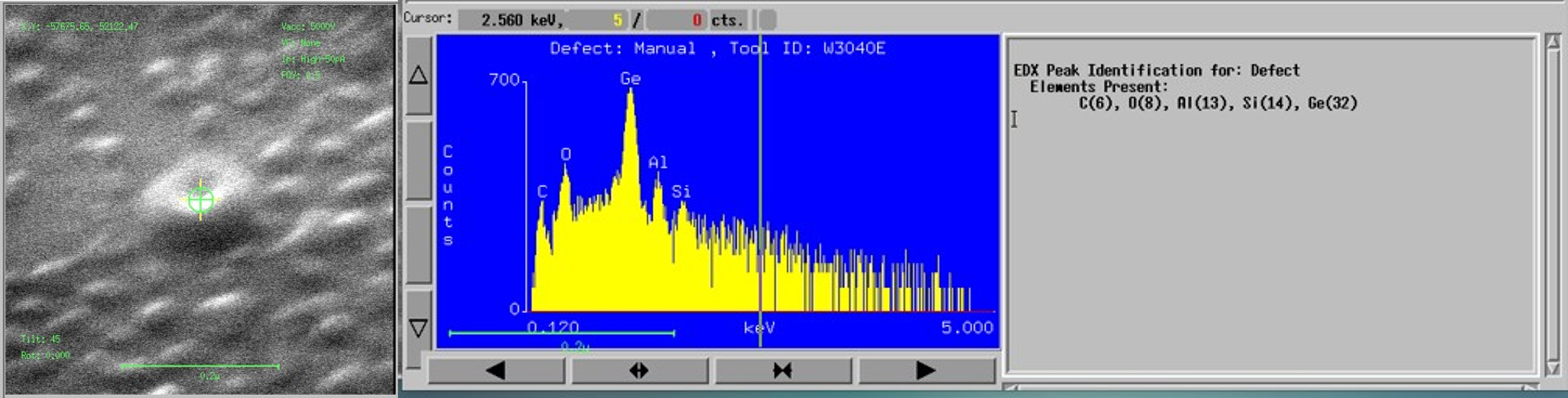


**Figure S2.** SEM-EDX at 5kV of bumps after leaving out ALD Al_2_O_3_ samples at ambient for 1 week.

*3D Atomic Force Microscopy (3D AFM)*

The 3D AFM measurements were done on the same waveguide structure of three GOS device wafers which were fabricated the same way. The roughness data was measured using Park Systems NX-3DM under non-contact head mode with head rotation 38° and scan size of 4 µm x 8 µm (128 lines x 4096 pixels). Fig. S3 shows the 3D imaging of the sidewall, the plot of the surface and sidewall roughness and the schematic of the line edge roughness (LER) measured along the height of the waveguide. The overall Rq measured was less than 3 nm as shown in Table 2.


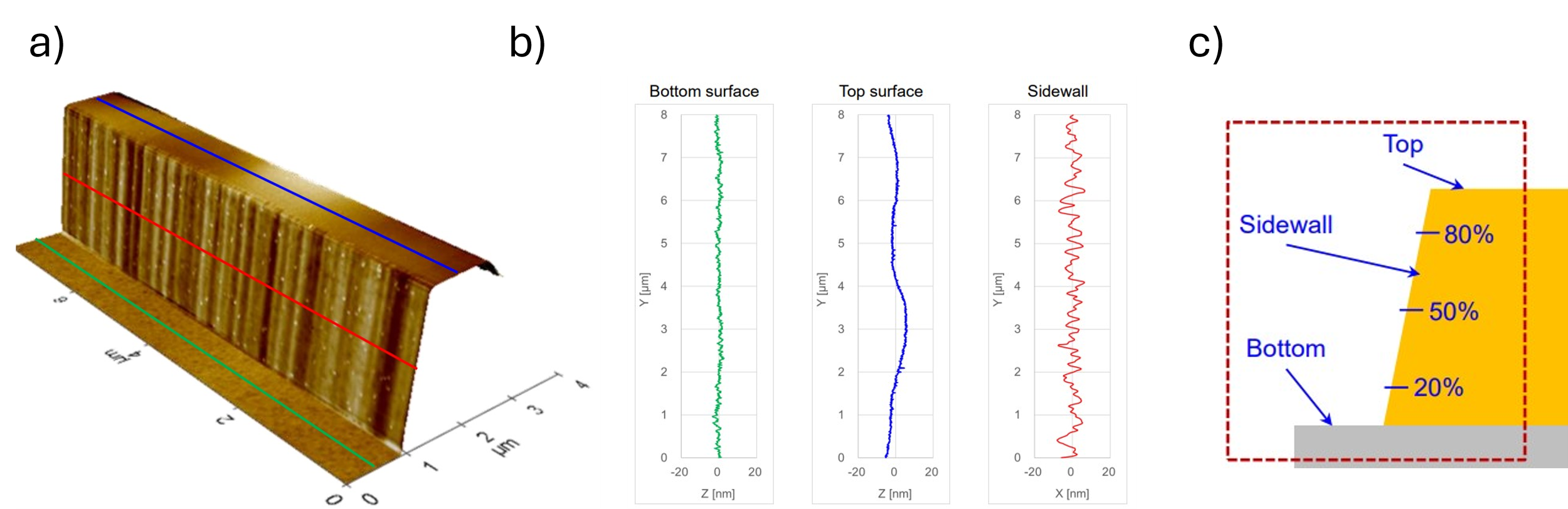


**Figure S3.** a) 3D imaging of one of the wafers’ GOS sidewall captured using 3D AFM with scan size of 4 µm x 8 µm. b) Line edge roughness (LER) plot of one of the wafers using scan size of 4 µm x 8 µm. c) Schematic of the LER data measured along the height of the waveguide.

**Table 2.** Sidewall roughness data collected across 3 wafers at the same waveguide structure using 3D AFM

| **Sample info** | | **Analysis result: *R*_q_ (nm)** | | | | |
| --- | --- | --- | --- | --- | --- | --- |
| Wafer No. | Structure | Bottom region | Top region | Sidewall | | |
|  |  |  |  | LER @ 80% | LER @ 50% | LER @ 20% |
| 1 | Waveguide | 0.832 | 2.870 | 2.288 | 2.301 | 2.056 |
| 2 |  | 1.077 | 0.818 | 1.462 | 1.593 | 1.653 |
| 3 |  | 1.561 | 0.553 | 1.326 | 1.488 | 1.556 |


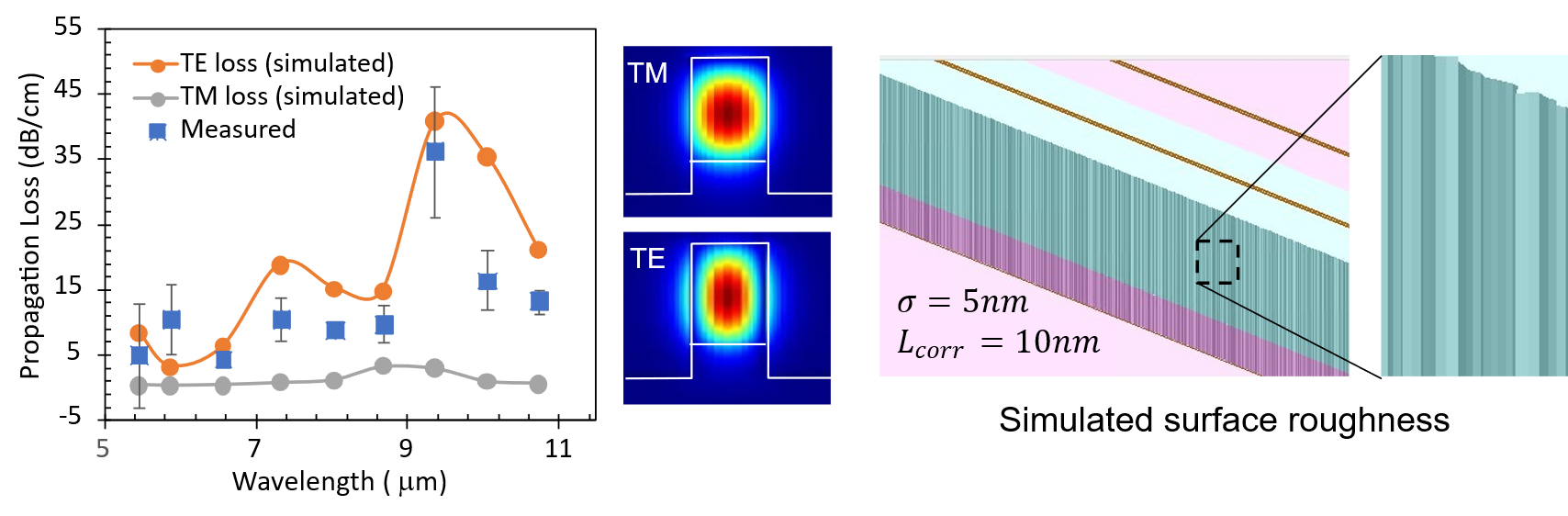


**Figure S4.** Simulation and measurement of pristine GOS waveguide from Ref [17]. The field profiles of the TM and TE modes are shown in the middle panels. The waveguide loss simulation is carried out by assuming line-edge-roughness (LER) of 5 nm and correlation length of 10 nm.
